# Supplementary material for: Generalizing population RT-qPCR cycle threshold values-informed estimation of epidemiological dynamics: Impact of surveillance practices and pathogen variability
Source: PLoS Comput Biol. 2025 Sep 29;21(9):e1013527. doi: 10.1371/journal.pcbi.1013527 (PMC12571249; doi:10.1371/journal.pcbi.1013527)
Supplement: Attachment — Submitted filename: response_CtRtsim_R1.docx [file pcbi.1013527.s003.docx]

**Reviewer #1:**

**Reviewer1Comm1:** Overall, this is a good manuscript that could be published in PLoS Computational Biology, following some additional analyses.

**Response:** Thank you for your supportive and helpful comments. Please see below for our detailed responses.

**Reviewer1Comm2:** My main concern is that the study does not currently motivate this new method over case-incidence Rt methods. I think that including the AUC of the incidence-based method as a comparison on the main figures would be useful. I also think that an additional main figure showing comparisons of both methods and the true value for each scenario (over time) would be highly valuable. Currently the only version of this in the main text is Figure 1A (some in supplementary without CIs). The Ct-based Rt estimate in this figure seems very uncertain and noisy and looks like a far worse metric than the slightly delayed Rt estimated using case-incidence. For the future (or this paper), it would useful if your method could include some statistical smoothing so there is less noise in daily estimates, this would lead to more reliable inferred trends over time, particularly when sample sizes are small.
**Response:** Thank you for your thoughtful suggestions. We agree that it is important to clearly motivate the value of the Ct-based Rt method, which is its timeliness, as highlighted in our previous work (1). We have therefore further emphasized this point in our revision, while clarifying that we do not intend Ct-based Rt as a replacement, but rather as a real-time supplementary metric for surveillance (in lines 96-99 and lines 235-241).

*“Considering the potential future risks of outbreaks posed by emerging or re-emerging pathogens(12), it is crucial to examine and refine new real-time surveillance metrics, including Ct-based methods, as supplementary tools that could provide additional and timely insights to traditional incidence-based* $R_{t}$ *for future applications.”*

*“By demonstrating that Ct-based Rt estimation methods remain accurate across diverse surveillance conditions and pathogen shedding patterns, our framework supports leveraging routinely collected RT-qPCR Ct data, which is often overlooked in surveillance, as a supplementary real-time metric alongside traditional incidence-based* $R_{t}$*. Such incorporation of Ct-based* $R_{t}$ *can offer timely and complementary insights into epidemic trends, which can be particularly useful for early outbreak surveillance in the future.”*

To enhance transparency, we have added a supplementary figure showing the trends of both Ct-based and incidence-based Rt estimates alongside the true Rt for each scenario over time (see Fig S4; referenced on lines 172-173).

Regarding smoothing, we have previously explored simple statistical smoothing (e.g., 3-, 7-, and 14-day rolling averages of Ct means and skewness), but found that it did not improve performance and sometimes reduced accuracy, possibly due to disruption of the temporal relationship between Ct distributions and Rt (see Supplementary Table 7 in Meng et al.(2), also attached below).

| **Table S7.** Area under the receiver operating characteristic curve (ROC) and directional mean absolute percentage error (MAPE) Ct-based 𝑅𝑡 and the incidence-based 𝑅𝑡 using different rolling Ct distributions. | | | | | | | | | |
| --- | --- | --- | --- | --- | --- | --- | --- | --- | --- |
|  | **Wave 4** | | | | **Wave 6** | | | | |
|  | **< 30^c^** | | **< 60^d^** | | **< 30^c^** | | **< 60^d^** | | |
|  | **AUC^a^** | **MAPE^b^** | **AUC^a^** | **MAPE^b^** | **AUC^a^** | **MAPE^b^** | **AUC^a^** | **MAPE^b^** |  |
| **Training period: wave 3 (6 Jul-31 Aug 2020)** | | | | | | | | | |
| **3-day rolling Ct** | 0.67 (0.6,0.75) | 0.29 | 0.67 (0.6,0.75) | 0.28 | 0.61 (0.52,0.7) | 0.24 | 0.6 (0.51,0.69) | 0.24 |  |
| **7-day rolling Ct** | 0.71 (0.63,0.78) | 0.30 | 0.71 (0.63,0.78) | 0.30 | 0.61 (0.52,0.7) | 0.24 | 0.59 (0.5,0.68) | 0.25 |  |
| **14-day rolling Ct** | 0.76 (0.68,0.83) | 0.34 | 0.74 (0.67,0.81) | 0.35 | 0.61 (0.52,0.7) | 0.25 | 0.59 (0.5,0.68) | 0.25 |  |
| **Training period: wave 4 (20 Nov - 19 Dec 2020)** | | | | | | | | | |
| **3-day rolling Ct** | 0.68 (0.6,0.75) | 0.30 | 0.67 (0.6,0.75) | 0.30 | 0.61 (0.52,0.7) | 0.22 | 0.6 (0.51,0.69) | 0.22 |  |
| **7-day rolling Ct** | 0.73 (0.65,0.8) | 0.32 | 0.72 (0.64,0.79) | 0.32 | 0.61 (0.52,0.7) | 0.22 | 0.59 (0.5,0.68) | 0.23 |  |
| **14-day rolling Ct** | 0.76 (0.69,0.83) | 0.35 | 0.74 (0.67,0.81) | 0.36 | 0.61 (0.52,0.7) | 0.23 | 0.59 (0.5,0.68) | 0.23 |  |
| **Training period: wave 5 (21 Feb -22 Mar 2022)** | | | | | | | | | |
| **3-day rolling Ct** | 0.67 (0.59,0.75) | 2.09 | 0.69 (0.61,0.77) | 2.07 | 0.53 (0.49,0.57) | 0.56 | 0.52 (0.49,0.56) | 0.57 |  |
| **7-day rolling Ct** | 0.62 (0.54,0.7) | 1.10 | 0.61 (0.53,0.69) | 1.05 | 0.53 (0.49,0.57) | 0.56 | 0.52 (0.49,0.56) | 0.58 |  |
| **14-day rolling Ct** | 0.57 (0.49,0.65) | 0.93 | 0.54 (0.47,0.62) | 0.87 | 0.53 (0.49,0.57) | 0.56 | 0.52 (0.49,0.56) | 0.58 |  |
| **Training period: wave 6 (28 Aug-26 Sep 2022)** | | | | | | | | | |
| **3-day rolling Ct** | 0.71 (0.64,0.77) | 0.29 | 0.71 (0.64,0.78) | 0.28 | 0.65 (0.58,0.73) | 0.23 | 0.65 (0.57,0.73) | 0.23 |  |
| **7-day rolling Ct** | 0.72 (0.65,0.79) | 0.30 | 0.71 (0.64,0.78) | 0.30 | 0.65 (0.58,0.73) | 0.24 | 0.65 (0.57,0.73) | 0.24 |  |
| **14-day rolling Ct** | 0.69 (0.62,0.76) | 0.33 | 0.68 (0.62,0.75) | 0.34 | 0.65 (0.58,0.73) | 0.24 | 0.65 (0.57,0.73) | 0.25 |  |
| **Training period: wave 7 (18 Dec 2022-16 Jan 2023)** | | | | | | | | | |
| **3-day rolling Ct** | 0.72 (0.65,0.8) | 0.32 | 0.71 (0.64,0.79) | 0.31 | 0.65 (0.56,0.74) | 0.24 | 0.64 (0.55,0.73) | 0.24 |  |
| **7-day rolling Ct** | 0.73 (0.66,0.81) | 0.32 | 0.72 (0.64,0.79) | 0.32 | 0.65 (0.56,0.74) | 0.24 | 0.64 (0.55,0.73) | 0.25 |  |
| **14-day rolling Ct** | 0.75 (0.68,0.82) | 0.36 | 0.71 (0.64,0.78) | 0.37 | 0.65 (0.56,0.74) | 0.24 | 0.63 (0.54,0.72) | 0.25 |  |
| a AUC: area under the receiver operating characteristic curve (ROC). | | | | | | | | | |
| b MAPE: mean absolute percentage error. | | | | | | | | | |
| c When daily Ct records was less than 30, we used rolling Ct values rather than daily Ct values to fit the model. | | | | | | | | | |
| d When daily Ct records was less than 60, we used rolling Ct values rather than daily Ct values to fit the model. | | | | | | | | | |

We therefore focus on daily estimates in our main analysis, but have expanded our discussion of these trade-offs and future directions for improved approaches in the revised manuscript (lines 296-302).

*“In addition, estimating* $R_{t}$ *from daily Ct distributions produces more variable estimates than incidence-based* $R_{t}$*, which typically applies a 7-day or 14-day smoothing window(3). Previous work found that simple statistical smoothing of Ct values did not improve performance and sometimes reduced accuracy, likely because it disrupted the temporal relationship between Ct distributions and* $R_{t}$*(11). Future methodological improvements that reduce daily fluctuations in population Ct values while preserving accurate and timely nowcasting could improve the utility of this approach.”*

**Reviewer1Comm3:** Given the relationship between Ct value distribution and incidence-based Rt estimates are used to parameterize the Ct-based model, can the Ct-based model ever perform better than using case data? Have you considered investigating model performance when you already have data quantifying viral shedding duration (so you don’t need to do a training period)?

**Response**: Thank you for the comment. In our current work, we use incidence-based Rt estimates to train the Ct-based model to leverage the temporal information in population-level Ct value distributions while keeping the model straightforward. We do not intend to propose Ct-based Rt as a replacement or to outperform incidence-based Rt estimates. Rather, our goal is to demonstrate that Ct-based Rt can serve as an additional, complementary surveillance metric. We agree that it would be possible to develop a fully independent Ct-based model that does not rely on incidence data for training, especially when data quantifying viral shedding duration can be available. However, our primary aim in this study was to establish the general consistency and feasibility of inferring epidemic progression from population-level Ct values. We now highlight this limitation and future direction in the revised Discussion (lines 310-312):

*“While our current approach uses incidence-based* $R_{t}$ *estimates to calibrate the Ct-based model, future work could develop fully independent Ct-based models that do not require case data for training.”*

**Reviewer1Comm4:** Additional analyses highlighting the value of this method and detailing when it performs better or worse than incidence-based methods can provide valuable details on when it is useful to implement/what policymakers should weight the estimates from each method during different periods. One potential analysis that could demonstrate the value of this methodology is when case testing gets capped following the pandemic (due to limited resources). Your model could still be fit to incidence-based methods during the first wave, and then could be shown to be effective at estimating Rt following the reduction in testing capacity, when incidence-based methods would be highly biased. This is similar to one of the scenarios that you have already performed analyses for (this could be extended to consider different testing rate caps/sample sizes)
**Response:** Thank you for this helpful comment. We agree that a key strength of Ct-based Rt estimation is its robustness in situations where incidence-based methods may be biased or unreliable, such as during limited testing capacity or changing surveillance practices. In our current study, we specifically analysed scenarios with capped daily case detection (scenario 5) and detection biases toward severe cases (scenarios 7 to 14), both of which reflect real-world challenges when surveillance systems are overwhelmed. Our results show that Ct-based Rt remains moderately to highly accurate in these contexts (Fig 1). For example, the difference in model accuracy between Ct-based and incidence-based Rt was minimal under limited detection capacity (i.e., scenario 5; see Fig S4B), demonstrating the value of Ct-based Rt as a complementary metric when case counts are incomplete.

In addition to highlighting scenarios where Ct-based Rt provides valuable information, our comparisons also reveal situations where its estimates may be less reliable, such as during abrupt changes in surveillance or when detection is heavily biased toward severe cases (Fig 1). We believe identifying these limitations is also important for understanding the method’s practical use.

While our study focused on a set of representative scenarios to balance both clarity and focus, we acknowledge the value of additional analyses, such as varying testing caps or sample sizes, as suggested. These extensions would be a promising avenue for future work. Because surveillance conditions can vary widely in real-world settings, we selected examples that best illustrate key challenges without expanding the scope of the study beyond our primary objectives. We also note that the robustness of the Ct-based method is supported by both simulation results and application to empirical data from Hong Kong and other settings(1-5). Nonetheless, we have further discussed the strengths and limitations of Ct-based Rt in our revised discussion (lines 244-253), and outlined relevant future direction in lines 306-308:

*“**As the method relies on the distribution of Ct values rather than absolute case counts, even limited or moderately biased detection can provide informative signals of epidemic changes, as observed in surveillance scenarios 5 and during the Omicron waves in Hong Kong when changes in testing and case ascertainment likely decreased the accuracy of incidence-based* $R_{t}$ *estimates (7, 11).* *However, dramatic shifts, such as a sudden bias in detecting only severe cases, which typically have longer shedding durations and lower Ct values, or delays in detecting these cases, can distort the learned Ct-*$R_{t}$ *relationship and reduce estimation accuracy, as observed in some of our simulated surveillance scenarios and during epidemic periods when sample representativeness changed or case numbers plateaued(11).”*

*“Additionally, expanding analyses or validating this method under more complicated and varying surveillance conditions in simulations or real-world settings will provide further insights and point to important directions for future work.”*

**Reviewer1Comm5:** Minor comments:
Abstract
“However, it remains unclear whether this approach can be broadly applied to other pathogens, sources of virologic test data, or surveillance strategies beyond those used during the pandemic, such as in Hong Kong”: I was confused by the “, such as in Hong Kong”

**Response:** Thank you for the comment and we have revised in lines 29-32 for clarification:

*“However, it remains unclear whether this approach can be broadly applied to other pathogens, sources of virologic test data, or surveillance strategies beyond those specifically implemented during the COVID-19 pandemic in Hong Kong.”*

**Reviewer1Comm6:** “Influenza A” which one? Throughout the paper the authors refer to influenza A. It would be useful to know if it is influenza A H3N2 or H1N1 and If not some comment on if they have similar viral shedding patterns.
**Response**: Thank you for the comment. We used parameter estimates representing influenza A as a group regardless of subtypes, as most of the top cited papers combined data from multiple subtypes (i.e., including both seasonal and pandemic, H3N2 and H1N1), and there was insufficient data for reliable subtype-specific modelling. Nonetheless, our review found no substantial differences in key parameters between influenza A subtypes, such as incubation period or duration of viral shedding. For example, both mixed influenza A strains and pandemic H1N1 typically have an incubation period of about 1.4 days(6, 7), and the mean duration of viral shedding is reported as 4.80 days with no significant difference between subtypes(8). We have clarified this in Supplementary page 3 and in the footnote of Table S3:

*“We used estimates representing influenza A regardless of subtypes due to insufficient data for subtype-specific modeling, and because relevant parameters did not differ substantially between subtypes (Table S3).”*

**Reviewer1Comm7:** The last paragraph of abstract could be rewritten to emphasize the findings of this work. Currently the final paragraph doesn’t mention that the models were in general accurate. It only mentions that some models exhibited lower accuracy. This will likely need to be edited based on findings of model performance between incidence-based and Ct-based methods (see major comment)
**Response:** We thank the reviewer for this suggestion. We have revised the final paragraph of the abstract to more clearly highlight the generally accurate performance of the models under various simulated scenarios and to clarify the findings regarding model performance (see lines 46-49 in the revised abstract):

*“Overall, our findings demonstrate that Ct-based* $R_{t}$ *estimation methods are generally accurate across diverse surveillance conditions and pathogen shedding patterns, supporting their practical use as a supplementary tool for timely transmission monitoring while highlighting limitations that warrant further consideration.”*

**Reviewer1Comm8:** Figure 1A – CT-based Rt seems all over the place compared to case incidence-based Rt? (see major comment)
**Response:** Thank you for this observation. The greater variability in Ct-based Rt estimates is mainly due to our use of daily Ct distributions, while incidence-based Rt was calculated with a 14-day smoothing window, resulting in smoother estimates. As shown in Figure 3 of Cori et al.(9), incidence-based Rt can also appear highly variable with shorter smoothing windows, particularly when case numbers are low. Thus, the observed fluctuations in Ct-based Rt are largely due to the choice of smoothing window and the smaller number of daily Ct samples in some periods. We have acknowledged and discussed this limitation in lines 296-298:

*“In addition, estimating* $R_{t}$ *from daily Ct distributions produces more variable estimates than incidence-based* $R_{t}$*, which typically applies a 7-day or 14-day smoothing window(3).”*

**Reviewer1Comm9:** Figure 3B – It would be useful to have more informative labels for “Pathogen X” so that the reader doesn’t have to refer back to Figure 1
**Response:** Thank you for the suggestion. We have revised Figure 3 to include informative labels specifying the shedding pattern for each pathogen, so that readers no longer need to refer back to Figure 1.

**Reviewer1Comm10:** Lines 104-160 read as Methods to me. I personally think this should be a methods section, but do not mind if this doesn’t change. The later methods section could be a supplementary methods section or moved up to join this section.
**Response:** Thank you for your feedback. Because our study involves extensive simulations, we aimed to provide an overview of the simulated scenarios upfront to help readers clearly understand the context of our results. We have revisited this section to make it more concise and focused on essential information for understanding the simulation setup, while retaining detailed methods in the Methods section.

**Reviewer1Comm11:** Discussion:
Lines 243-245. This links back to my major comment on the manuscript. The final sentence of this paragraph is not motivated by the findings of the paper. There is currently no description as to why this method would be used instead of incidence-based Rt estimation. This is particularly true given the relationship between Ct value distribution and incidence-based Rt estimates are used to parameterize the Ct-based model.
**Response:** Thank you for this comment. We have revised the discussion to better emphasize our main findings and to clarify that we do not aim to replace traditional incidence-based Rt estimation. Instead, we propose Ct-based Rt as a real-time metric to provide more timely and complementary insights into epidemic trends. These revisions can be found in lines 235-241 of the revised manuscript:

*“By demonstrating that Ct-based* $R_{t}$ *estimation methods remain accurate across diverse surveillance conditions and pathogen shedding patterns, our framework supports leveraging routinely collected RT-qPCR Ct data, which is often overlooked in surveillance, as a supplementary real-time metric alongside traditional incidence-based* $R_{t}$*. Such incorporation of Ct-based* $R_{t}$ *can offer timely and complementary insights into epidemic trends, which can be particularly useful for early outbreak surveillance in the future.”*

**Reviewer1Comm12:** Lines 275-277. Very interesting ideas for future applications. Given the model here used case incidence-based Rt estimates to infer the relationship between Rt and Ct would additional data be needed for this extension?
**Response:** Thank you for this insightful question. Applying the Ct-based Rt estimation framework to H5N1 or other novel settings would indeed require additional data, including systematic collection of Ct values from at-risk or infected animals and exposed individuals. Although estimating transmission dynamics using incidence-based Rt may be challenging in these settings due to limited case reporting, tracking Ct values could still offer valuable insights into transmission dynamics due to their temporal relationship with infection. We have expanded the discussion on this in lines 276-278:

*“… despite challenges such as species-specific variability, asymptomatic shedding(19-21) and the need for additional surveillance data, particularly systematic Ct value collection from at-risk or infected poultry and exposed individuals.”*

**Reviewer1Comm13:** Methods
Line 318 “symptomatic cases were detected at a fixed probability of 25%”. I assume this means that daily cases were pulled from a Binomial distribution ~B(N=infections, p=0.25)? Perhaps this could be stated for added clarity.
**Response:** Thank you for your comment. We have revised lines 339-343 for clarification as suggested.

*“For the typical scenario of stable case detection similar to the first four COVID-19 waves in Hong Kong(25) (i.e., scenario 1), daily detected symptomatic cases were simulated by drawing from a binomial distribution:* $Binomial(N=number of new infection, p=0.25)$*. To reflect small daily fluctuations in detection probability, noise (e.g., ±0.5%) was added around the mean probability of 25%.”*

**Reviewer1Comm14:** Line 390. The language is a bit unclear to me on how training periods were selected? It is more clear in the supplementary methods so maybe some language there can be added here.
**Response:** Thank you for your comment. We have clarified our approach to selecting training periods in lines 414-420, incorporating language from the Supplementary Methods as suggested.

*“To establish a generic training period applicable to all scenarios, we systematically evaluated different candidate training periods during the first simulated wave. Specifically, we varied the start dates and durations of these potential training periods, fitted regression models to each, and compared their adjusted R-squared values. For each scenario, the training period that produced the highest adjusted R-squared was selected as the best-performing period (see* ***Supplementary Methods****).”*

**Reviewer1Comm15:** Supplementary methods:
Estimation of incidence-based Rt: In estimating Rt using incidence-based methods you have assumed that delay distributions and incubation period were known exactly. In future work you could consider that these introduce biases that Ct-based methods could overcome (if you don’t train the model on incidence based methods).

**Response:** Thank you for your insightful comment. We agree that it is important to investigate how Ct-based approaches may help address biases arising from uncertainty in delay distributions and incubation periods in incidence-based Rt estimation, particularly when models are not trained on incidence data. We have added this point to lines 310-315 in the Discussion section as a potential direction for future research.

*“While our current approach uses incidence-based* $R_{t}$*estimates to calibrate the Ct-based model, future work could develop more generative or fully independent Ct-based models that do not require case incidence data for training. Because incidence-based* $R_{t}$ *estimation depends on delay distributions and incubation periods that are often uncertain and may introduce bias, Ct-based methods developed independently of incidence data could help overcome these limitations.”*

**Reviewer #2:**

**Reviewer2Comm1:** This study evaluated the utility of cycle threshold (Ct) value-based estimation of the reproductive number (Rt) under varying surveillance conditions, epidemiological scenarios, and viral shedding kinetics. The method demonstrated robustness to variation in case ascertainment, provided that ascertainment rates remained stable over time and were not biased by disease severity. In terms of viral kinetics, accurate estimation was achieved when the viral load peaked before symptom onset, resulting in a monotonic shedding pattern that more reliably corresponds to time since infection. These findings are largely intuitive: stable detection minimally affects incidence-based Rt estimation, and a monotonic relationship between viral load and infection time allows Ct values to serve as a temporal marker. The authors’ simulations comprehensively support these conclusions. However, the manuscript would benefit from a more detailed discussion of the simulation assumptions, particularly regarding the detection mechanisms and their impact on the representativeness of population-level Ct distributions.

**Response:** Thank you for your supportive comment. While we have described our simulation setup and key assumptions in both the Results and Methods sections (particularly ***“Simulation of epidemic waves, detected individuals, and viral loads”*** and the Supplementary Methods), we agree that further discussion of these assumptions would strengthen the manuscript. We have now expanded the Discussion to address our assumptions regarding detection mechanisms and their impact on the representativeness of population-level Ct distributions (see lines 282–287):

*“Although we have analyzed various surveillance practices and circulating pathogens, our assumptions about detection mechanisms, including who is tested and when, may not fully capture real-world complexities. Additionally, factors such as co-circulating virus subtypes and heterogeneous immune backgrounds can further affect the representativeness of observed Ct distributions and the accuracy of resulting* $R_{t}$ *estimates.”*

**Reviewer2Comm2:** Major Comments:
1. The manuscript highlights the robustness of Ct-based Rt estimation under surveillance variability, but it would be helpful to see a more direct comparison with conventional incidence-based methods. Incidence-based Rt can also be biased, especially under delayed or inconsistent reporting. Under what conditions does the Ct-based approach clearly outperform case-based methods? Clarifying this point would improve the practical guidance for choosing between methods in real-world settings.
**Response:** Thank you for your helpful suggestion. We have added a supplementary figure directly comparing the accuracy of incidence-based and Ct-based Rt estimates across simulated surveillance scenarios. Our results show that while incidence-based Rt is generally accurate, its performance declines under limited detection capacity (scenario 5), where the difference in AUC between the two methods narrows (Fig S4). This suggests that Ct-based Rt may be particularly valuable when case detection is incomplete or variable, as also supported by our previous work(1) and further highlighted in lines 244-248:

*“As the method relies on the distribution of Ct values rather than absolute case counts, even limited or moderately biased detection can provide informative signals of epidemic changes, as observed in surveillance scenarios 5 and during the Omicron waves in Hong Kong when changes in testing and case ascertainment likely decreased the accuracy of incidence-based* $R_{t}$ *estimates (7, 11).”*

Importantly, as discussed in our previous work(1), the Ct-based approach improves timeliness by using the population-level Ct distribution from samples collected on a given day, rather than depending on delayed case reporting. This helps address right censoring, where cases that are infected but not yet detected are missed by incidence-based methods, which is particularly important in settings with reporting delays or backlogs. We have further highlighted this in the introduction in lines 75-81:

*“To provide an alternative method for real-time* $R_{t}$ *estimation, inference models have integrated population-level viral load data measured through cycle threshold (Ct) values from reverse-transcription quantitative polymerase chain reaction (RT-qPCR)(6, 7). This approach, validated in simulations using random Ct samples(6) and applied in Hong Kong’s epidemic waves with symptom and contact-tracing-based surveillance(7), leverages Ct value data collected on a given day to provide earlier insights into transmission dynamics.”*

We also note that the accuracy of incidence-based Rt was higher than our current simplified and exploratory Ct-based Rt estimation, as the incidence-based method uses more advanced and comprehensive analytical techniques, whereas our Ct-based approach is intentionally kept simple and straightforward for this initial exploration. We have clarified throughout the manuscript that the Ct-based approach is proposed as a complementary and more timely surveillance metric, rather than a replacement for incidence-based methods. This clarification is reflected in multiple sections, including the revised discussion in lines 235-241:

*“By demonstrating that Ct-based* $R_{t}$ *estimation methods remain accurate across diverse surveillance conditions and pathogen shedding patterns, our framework supports leveraging routinely collected RT-qPCR Ct data, which is often overlooked in surveillance, as a supplementary real-time metric alongside traditional incidence-based* $R_{t}$*. Such incorporation of Ct-based* $R_{t}$ *can offer timely and complementary insights into epidemic trends, which can be particularly useful for early outbreak surveillance in the future.”*

**Reviewer2Comm3:** 2. The simulations assume that only symptomatic individuals are eligible for detection and Ct measurement, with fixed detection probabilities and delays. How sensitive is the accuracy of Ct-based Rt estimation to the assumed symptomatic ratio? Since asymptomatic cases are common and often undetected (and contribution to transmission and viral dynamics might be different), it would be useful to understand how their exclusion affects the generalizability and robustness of the method.

**Response:** Thank you for your comment. We focused on symptomatic individuals because they are the primary group captured by typical targeted surveillance systems, such as those implemented during COVID-19 and influenza seasons. In our previous work using empirical data from Hong Kong, we specifically explored the impact of varying proportion of detected symptomatic cases and found that the accuracy of Ct-based Rt estimation was not substantially affected, as symptomatic cases are generally those most likely to be detected and included in analyses(1).

We acknowledge the important role of asymptomatic individuals in transmission for pathogens like SARS-CoV-2 and influenza. However, asymptomatic cases are generally difficult to identify without large-scale testing or intensive contact tracing, which are often not feasible in most real-world settings. Including Ct data from asymptomatic individuals and evaluating the method in settings with regular, comprehensive testing (e.g., constrained environments like elderly care homes) would be valuable because Ct values obtained in these contexts may more accurately reflect time since infection and are less influenced by detection practices(10). However, simulating these scenarios would require a fundamentally different setup and is beyond the scope of our current focus on typical targeted (syndromic) surveillance. We agree this is an important area for future research and have added a discussion of these points in lines 302-305:

*“While our analysis focused on symptomatic individuals typically detected through targeted and syndromic surveillance, future research should also explore incorporating Ct values from asymptomatic cases in settings with more comprehensive and universal testing.”*

**References:**

1.	Lin Y, Yang B, Cobey S, Lau EHY, Adam DC, Wong JY, et al. Incorporating temporal distribution of population-level viral load enables real-time estimation of COVID-19 transmission. Nat Commun. 2022;13(1):1155.

2.	Meng Y, Lin Y, Xiong W, Lau EHY, Ho F, Wong JY, et al. Effective Real-time Transmission Estimations Incorporating Population Viral Load Distributions Amid SARS-CoV-2 Variants and Preexisting Immunity. J Infect Dis. 2025;231(3):684-91.

3.	Ahuja V, Bowe T, Warnock G, Pitman C, Dwyer DE. Trends in SARS-CoV-2 cycle threshold (Ct) values from nucleic acid testing predict the trajectory of COVID-19 waves. Pathology. 2024.

4.	Musalkova D, Piherova L, Kwasny O, Dindova Z, Stancik L, Hartmannova H, et al. Trends in SARS-CoV-2 cycle threshold values in the Czech Republic from April 2020 to April 2022. Sci Rep. 2023;13(1):6156.

5.	Sharmin M, Manivannan M, Woo D, Sorel O, Auclair JR, Gandhi M, et al. Cross-sectional Ct distributions from qPCR tests can provide an early warning signal for the spread of COVID-19 in communities. Front Public Health. 2023;11:1185720.

6.	Lessler J, Reich NG, Cummings DA, Nair HP, Jordan HT, Thompson N. Outbreak of 2009 pandemic influenza A (H1N1) at a New York City school. N Engl J Med. 2009;361(27):2628-36.

7.	Lessler J, Reich NG, Brookmeyer R, Perl TM, Nelson KE, Cummings DA. Incubation periods of acute respiratory viral infections: a systematic review. Lancet Infect Dis. 2009;9(5):291-300.

8.	Carrat F, Vergu E, Ferguson NM, Lemaitre M, Cauchemez S, Leach S, et al. Time lines of infection and disease in human influenza: a review of volunteer challenge studies. Am J Epidemiol. 2008;167(7):775-85.

9.	Cori A, Ferguson NM, Fraser C, Cauchemez S. A new framework and software to estimate time-varying reproduction numbers during epidemics. Am J Epidemiol. 2013;178(9):1505-12.

10.	Hay JA, Kennedy-Shaffer L, Kanjilal S, Lennon NJ, Gabriel SB, Lipsitch M, et al. Estimating epidemiologic dynamics from cross-sectional viral load distributions. Science. 2021;373(6552).
